# The utility of peritoneal drains in patients with perforated appendicitis

**DOI:** 10.1186/s40064-015-1154-9

**Published:** 2015-07-24

**Authors:** Martinus A Beek, Tim S Jansen, Jelle W Raats, Eric L L Twiss, Paul D Gobardhan, Eric J H van Rhede van der Kloot

**Affiliations:** Department of Surgery, Amphia Hospital, Molengracht 21, 4818 CK Breda, The Netherlands; Department of Surgery, VU University Medical Center, De Boelelaan 1117, 1081 HV Amsterdam, The Netherlands

**Keywords:** Perforated appendicitis, Peritoneal drainage, Complications, Peritoneal drain

## Abstract

**Background:**

Intra-abdominal abscesses are the most common complication after perforated appendicitis and remain a significant problem ranging in incidence from 14 to 18%. Drainage following appendectomy is usually determined by whether the underlying appendicitis is simple or complicated and largely determined by the surgeons’ belief, based on expertise or personal opinion. In this report we discuss the results of patients diagnosed with peritoneal drainage, treated with or without a peritoneal drain.

**Patients and methods:**

A retrospective study of patients diagnosed with perforated appendicitis having surgery was performed. Patients diagnosed with perforated appendicitis treated with a peritoneal drain and patients treated without a peritoneal drain. Both groups were evaluated in terms of complications: intra-abdominal abscess, re-intervention, readmission and duration of hospital stay.

**Results:**

199 patients diagnosed with perforated appendicitis underwent appendectomy. 120 patients were treated without a peritoneal drain and 79 patients with a peritoneal drain. Thirty-one (26%) patients from the group without a peritoneal drain had a re-intervention compared to 9 (11%) in the group with a peritoneal drain (*p* = 0.013). Overall complications and readmission were also significantly lower in patients treated with a peritoneal drain.

**Conclusion:**

A peritoneal drain seems to reduce overall complication rate, re-intervention rate and readmission rate in patients treated with perforated appendicitis.

## Background

The lifetime risk of appendicitis is 9% for men and 7% for women (Addiss et al. 1990). Acute appendicitis is a common disease with a peak incidence between 15 and 30 years. Acute appendicitis remains the most common general surgical emergency seen in most hospitals and the most common cause of acute abdomen requiring surgical intervention.

In contrast to acute uncomplicated appendicitis, the perforated form is related to an increased risk of post-operative complications and is related to adverse outcome. Intra-abdominal abscesses are the most common complication after perforated appendicitis and remain a significant problem ranging in incidence from 14 to 18% (Fraser et al. 2010; St Peter et al. 2008a, b). In contrast to patients with acute uncomplicated appendicitis, reporting incidence form 1–2% (St Peter et al. 2008b).

Peritoneal drainage is widely used by surgeons in current clinical practice. Leaving a drain in the peritoneal cavity in case of perforated appendicitis, intra-abdominal abscess formation after appendectomy could potentially be prevented (Curran and Muenchow 1993; Fishman et al. 2000; Lund and Murphy 1994). Retention of possible contaminated intra-abdominal fluids could be drained timely.

Nevertheless, routine peritoneal drainage after appendectomy in case of perforated appendicitis remains topic of debate (Narci et al. 2007). Many surgeons use peritoneal drains selectively now (Dandapat and Panda 1992; Schwartz et al. 1983; Yamini et al. 1998), although others recommend routinely use of drains in case of perforated appendicitis (Curran and Muenchow 1993; Fishman et al. 2000; Lund and Murphy 1994).

In addition, the impact of an abscess on patient outcome is tremendous and directly increases hospital stay and hospital costs (Gasior et al. 2013). Therefore, prevention of intra-abdominal abscesses after appendectomy is of major importance.

Although many studies have reported outcomes after appendectomy concerning perforated appendicitis, there is still major controversy regarding the optimal management of perforated appendicitis. In this study we report results of patients operated for perforated appendicitis, treated with or without peritoneal drainage in current clinical practice.

## Patients and methods

### Patients

All patients treated in our hospital for acute appendicitis between January 2011 and August 2013 enrolled the study cohort. Patients with uncomplicated appendicitis and patients with a malignancy (after pathological examination) were excluded (n = 1,029). A total of 199 patients diagnosed with perforated appendicitis were included for further analysis.

### Diagnosis

All patients were pre-operatively examined by the surgeon on call. The diagnosis of appendicitis was made by the attending surgeon according to the guidelines of the Association of Surgeons of the Netherlands (Heelkunde 2010). If necessary, additional ultrasonography or multislice computed tomography (CT, Siemens Definition scanner, Siemens, Munich, Germany) was performed to confirm diagnosis of acute appendicitis. Diagnosis of perforated appendicitis was made intra-operatively.

### Treatment

All patients received preoperative antimicrobial prophylaxis consisting of intravenous Cefazoline and Metronidazole however, in patients with a known allergic reaction of one of these antibiotics a combination of Clindamycin and Tobramycin was prescribed. General anesthesia was performed in all patients. The operating surgeon decided whether laparoscopic or open appendectomy was performed, based on surgeon specific experience and preferences. Peritoneal lavage with warmed with isotonic saline was performed after appendectomy.

Leaving an intra-peritoneal drain (Silicone- or Redon drain) after appendectomy was decided by the performing surgeon based on the observed operative contamination and expertise.

In all patients, the abdominal fascia was closed, in some selected cases the dermis was approximated or left open. Postoperatively intravenous antibiotics were prescribed in all included patients for at least 3 days following our hospital protocols. Drains were removed after at least 24 h based on the production and aspect of the drained fluid.

### Outcome

Patients were classified into two groups. The first group consisted of patients diagnosed with perforated appendicitis treated with peritoneal drainage. The second group consisted of patients diagnosed with perforated appendicitis treated without peritoneal drainage.

Complications were identified and categorized in the following groups: overall complications, re-interventions, duration of hospital stay and readmissions. The duration of a readmission was included in the hospital stay calculation. Overall complications were defined as wound infection, intra-abdominal abscess formation, post-operative abdominal pain and stump leakage. Post-operative abdominal pain was defined as abdominal complains after surgery requiring prolonged clinical observation or additional biochemistry or radiological tests.

Re-interventions were defined as percutaneous drainage, re-laparoscopy/laparotomy, transrectal drainage and prolonged use of intravenous antibiotics (>3–5 days).

### Statistical analysis

Statistical analyses were performed using SPSS, version 21.0 (SPSS, Inc., Chicago, USA). Chi-square analysis was performed to evaluate proportional differences between the two groups. Mann–Whitney U test was performed for continues data. P values of ≤0.05 were considered significant.

### Ethics approval

Ethical approval for this study was provided by the local ethical committee of the Amphia Hospital.

## Results

Between January 2011 and August 2013 a total of 199 patients were diagnosed with perforated appendicitis and underwent appendectomy. A total of 79 (40%) patients were included in the group with a peritoneal drain and 120 (60%) patients in the group without a peritoneal drain. Between the groups no significant difference in age, gender and type of operation was observed (Table 1). There were 21 different operating surgeons and 3 of them never left an intra-peritoneal drain after appendectomy.

**Table 1:** Baseline characteristics of patients with perforated appendicitis

	Drain *n* = 79	No drain *n* = 120	*P* value
Gender (%)	44 (56)	63 (53)	0.658^a^
Age (range)	37 (6–83)	33 (3–82)	0.226^b^
Type of operation (%)			
Laparoscopic	42 (53)	82 (68)	
Open	18 (23)	21 (18)	
Conversion	19 (24)	17 (14)	0.082^a^

^a^Chi-square.

^b^Mann–Whitney U test.

### Overall complications

Overall, 55 patients developed a complication after surgery, 15 (19%) in the group with a peritoneal drain and 40 (33%) in the group without a peritoneal drain (*p* = 0.027). In the group without a peritoneal drain, post-operative abdominal pain was less frequently observed (0%) compared to the group with a peritoneal drain (15%; *p* = 0.004). No differences were observed between both groups in stump leak, wound infections or other complications (e.g. ileus, respiratory insufficiency, myocardial infarction or hospital acquired pneumonia). Data concerning complications are shown in Table 2.

**Table 2:** Complications of patients with perforated appendicitis having peritoneal drainage or not

	Drain *n* = 79 (%)	No drain *n* = 120 (%)	*P* value
Wound infection	2 (3)	4 (3)	1.000
Intra-abdominal abscess	5 (6)	18 (15)	0.061
Stump leakage	1 (1)	0 (–)	0.397
Post-operative abdominal pain	0 (–)	12 (15)	0.004
Other complications^a^	7 (9)	6 (5)	0.380
*Overall complications*	*15 (19)*	*40 (33)*	*0.027*

*P* value calculated with Chi-square test.

^a^Other complications concerned postoperative ileus, pulmonary embolism, urinary tract infection, delirium, pleural effusion, pneumonia.

### Intra-abdominal abscess

In the group with a peritoneal drain 5 patients (6%) developed an intra-abdominal abscess post operatively. In the group treated without a peritoneal drain 18 patients (15%) developed an intra-abdominal abscess after appendectomy. No statistical difference was observed between both groups (*p* = 0.061). Data are shown in Table 3.

**Table 3:** Outcomes of all operated patients who had a drain placed compared to those who did not

	Drain *n* = 79 (%)	No drain *n* = 120 (%)	*P* value
Re-intervention	9 (11)	31 (26)	0.013
Percutaneous drainage	1 (1)	10 (8)	0.033
Laparoscopy	0 (–)	2 (2)	0.519
Laparotomy	4 (5)	6 (5)	0.984
Transrectal drainage	1 (1)	2 (2)	1.000
Prolonged intravenous antibiotics^b^	3 (4)	11 (9)	0.147
Readmissions	4 (5)	19 (16)	0.020
Median duration of hospitalization (IQR)	5 (3)	5 (3)	0.643^a^

*IQR* Interquartile Range.

*P* value calculated with Chi-square test, unless otherwise specified.

^a^Mann–Whitney U test.

^b^Prolonged intravenous antibiotics was defined as >3–5 days.

### Re-interventions

Re-interventions were more observed in the patients treated without a peritoneal drain (26%) compared to patients with a peritoneal drain (11%; *p* = 0.013). Percutaneous drainage was more performed in patients treated without a peritoneal drain (8%) vs. patients with a peritoneal drain (1%; *p* = 0.033). No significant differences were observed between both groups in performed re-laparoscopy/laparotomies, transrectal drainage or prolonged use of intravenous antibiotics. Data concerning re-interventions are shown in Table 3.

### Readmissions and hospital stay

Readmissions were more frequent observed in the patients treated without a peritoneal drain (16%) vs. the patients treated with a peritoneal drain (5%; *p* = 0.020). No significant difference was found concerning duration of hospital stay between both groups. Data are presented in Table 3.

## Discussion

Although there is consensus about the aetiology of appendicitis, diagnosis and optimal treatment of this disorder are still under debate. In current literature, there is controversy concerning the use of a peritoneal drain in patients after treatment of perforated appendicitis (Narci et al. 2007).

Drainage following appendectomy is usually determined by whether the underlying appendicitis is simple or complicated and largely determined by the surgeons’ belief, based on expertise or personal opinion (Dandapat and Panda 1992; Schwartz et al. 1983; Yamini et al. 1998). However, some investigators recommend routine use of drains in case of perforated appendicitis (Curran and Muenchow 1993; Fishman et al. 2000; Lund and Murphy 1994). Evidence to guide this clinical decision is scarce, often outdated and based on small numbers (Dandapat and Panda 1992; Magarey et al. 1971; Haller et al. 1973; Greenall et al. 1978; Stone et al. 1978). Some authors suggest that the use of peritoneal drains increases work load for nursing staff and doctors (Tander et al. 2003). In this study, re-interventions and readmission is significantly higher in patients treated without a peritoneal drain.

In our study, 15% of the treated patients without a peritoneal drain developed an intra-abdominal abscess after appendectomy. If a peritoneal drain was used, abscess formation was reduced to 5 of 79 patients (6%; *p* = 0.061). Our reported data are comparable with previous studies (Dandapat and Panda 1992). Based on these data, additional studies need to include 140 subjects in the group without a peritoneal drain and 140 subjects in the group with a peritoneal drain to be able to reject the null hypothesis that the failure rates for experimental and control subjects are equal with a probability (power) of 0.8. The Type I error probability associated with this test of the null hypothesis is 0.05.

Despite the absence of statistical significance in this study, re-interventions and readmissions were lower in the patients treated with a peritoneal drain after appendectomy in case of perforated appendicitis. This could be explained by the fact that numbers were relatively small in our selected patient population. However, the observed adverse outcomes in patients treated without a peritoneal drain highlights clinical importance. As prolonged hospital stay and re-interventions could be averted, possible preventive actions should be considered, including routine peritoneal drainage.

Therefore, additional studies will have to answer the question whether routine peritoneal drainage may improve the outcome in patients in the context of perforated appendicitis.

In summary, our findings suggest that the use of a peritoneal drain reduces complications and readmission rate in patients with perforated appendicitis. Due to the shortcomings of the retrospective design of this study, recommendations cannot be made. Ideally, treatment with or without peritoneal drainage should be investigated in a randomized trial including a multivariate analysis.
